# Synthesis and crystal structure of a mixed-metal 3D coordination polymer poly[[bis­(μ_5_-anthra­quinone-1,8-di­sulfonato-κ^5^*O*:*O*′:*O*′′:*O*′′′:*O*′′′′)di-μ_2_-aqua-κ^4^*O*:*O*-tetra­aqua­copper(II)disodium] dihydrate]

**DOI:** 10.1107/S2056989026003956

**Published:** 2026-05-15

**Authors:** Xuan-Yi Chen, Jia Wei, Juan He, Hui-Lei Gao, Xu-Dong Chen, Gan Xu

**Affiliations:** a School of Chemistry and Materials Science Nanjing Normal University,Nanjing 210023 People’s Republic of China; Harvard University, USA

**Keywords:** anthra­quinone-1,8-di­sulfonic acid, coordination polymer, hydrogen bonding, crystal structure

## Abstract

Metal–organic hybrid materials have attracted a great deal of research inter­est due to their potential applications in the field such as gas storage and separation, heterogeneous catalysis, chemical/biological sensing and detection, energy transfer and photocatalysis, *etc*. The rational design and synthesis of organic ligands has proven to be an effective strategy in fabricating desired structures with given properties. Anthra­quinone-1,8-di­sulfonic acid (1,8-H_2_AQDS, C_8_H_14_O_8_S_2_) has been used to construct the title complex, {[Na_2_Cu(1,8-AQDS)_2_(H_2_O)_6_]·2H_2_O}_*n*_, by means of half-neutralization with NaOH followed by assembly with Cu(NO_3_)_2_·3H_2_O. This mixed-metal coordination polymer exhibits a three-dimensional pillar-layered framework structure with the 1,8-AQDS^2−^ ligand adopting a *μ_5_*-bridging mode, including a coordinated carbonyl group binding with Na^+^ cation. The Na^+^ and Cu^2+^ cations both exhibit a distorted octa­hedral coordination environment, in which the Na^+^ coordination sphere is more irregular stretched. The 1,8-substituted bulky sulfonate groups exert a strong stereo effect to the inter-positioned carbonyl group and lead to the bending of the 1,8-AQDS^2−^ ligand into a butterfly conformation. Both coordinated and solvent water mol­ecules are involved in O—H⋯O hydrogen bonding, which further consolidates the three-dimensional coordination framework.

## Abstract 2

A Na^I^–Cu^II^ mixed-metal three-dimensional coordination polymer based on anthra­quinone-1,8-di­sulfonate has been synthesized and characterized by single-crystal X-ray diffraction, infrared spectroscopy and thermogravimetric analysis. The complex exhibits a three-dimensional pillar-layered coordination framework with rich hydrogen-bonding motifs in the solid state.

## 1. Chemical context

Metal–organic hybrid materials exhibiting versatile topologies and fascinating structural motifs have attracted a great deal of research inter­est in the past two decades. Much effort has been devoted to the design and synthesis of coordination complexes with desired structural features, which are targeted at providing different functionalities that have potential applications in areas such as gas storage and separation (Murray *et al.*, 2009 ▸), heterogeneous catalysis (Lee *et al.*, 2009 ▸), chemical and biological sensing and detection (Hu *et al.*, 2014 ▸), energy transfer and photocatalysis (Zhang & Lin, 2014 ▸), *etc*. The rational design and synthesis of organic ligands as basic building blocks has been one of the most efficient strategies in constructing metal–organic hybrid complexes with various architectures. The exploration of versatile rigid or flexible organic ligands with various coordinating groups is therefore one of the central themes in this research area. A ligand with sulfonate groups is one of the inter­esting types of building blocks in this family. Normally, under hydrous conditions, sulfonate ligands and metal ions tend to form layered structures with hydrated metal cations and sulfonate ligands as alternate sheets that are paired ionically (Shimizu *et al.*, 2009 ▸). When pillaring ligands are used, the compact layered packing will be inter­rupted and it will result in pillared-layered structures with some porosities (Shimizu *et al.*, 2009 ▸). Another typical feature of the sulfonate ligands is that sulfonate anions have a high affinity in forming hydrogen-bonding with aqua ligands, ammonia and solvent water mol­ecules, leading to better stability of the supra­molecular structure and even hydrogen-bonded frameworks with permanent porosity (Dalrymple & Shimizu, 2007 ▸). Along these lines, the anthra­quinone­disulfonate ligands have in recent years attracted quite a lot of research inter­est in building up metal–organic hybrid complexes, with the emphasis being put on anthra­quinone-2,5-di­sulfonate and anthra­quinone-2,6-di­sulfonate (D’Vries *et al.*, 2012 ▸; Gándara *et al.*, 2012 ▸; Fu *et al.*, 2011 ▸; Wang *et al.*, 2014 ▸; Hou *et al.*, 2012 ▸; Zhang *et al.*, 2011 ▸; Platero-Prats *et al.*, 2011 ▸). However, metal complexes of anthra­quinone-1,8-di­sulfonate (1,8-AQDS^2−^) remain unexplored to date. In this paper, we report a mixed-metal coordination polymer {[Na_2_Cu(H_2_O)_6_(1,8-AQDS)_2_]·2H_2_O}_*n*_ (**1**) based on anthra­quinone-1,8-di­sulfonate ligand.
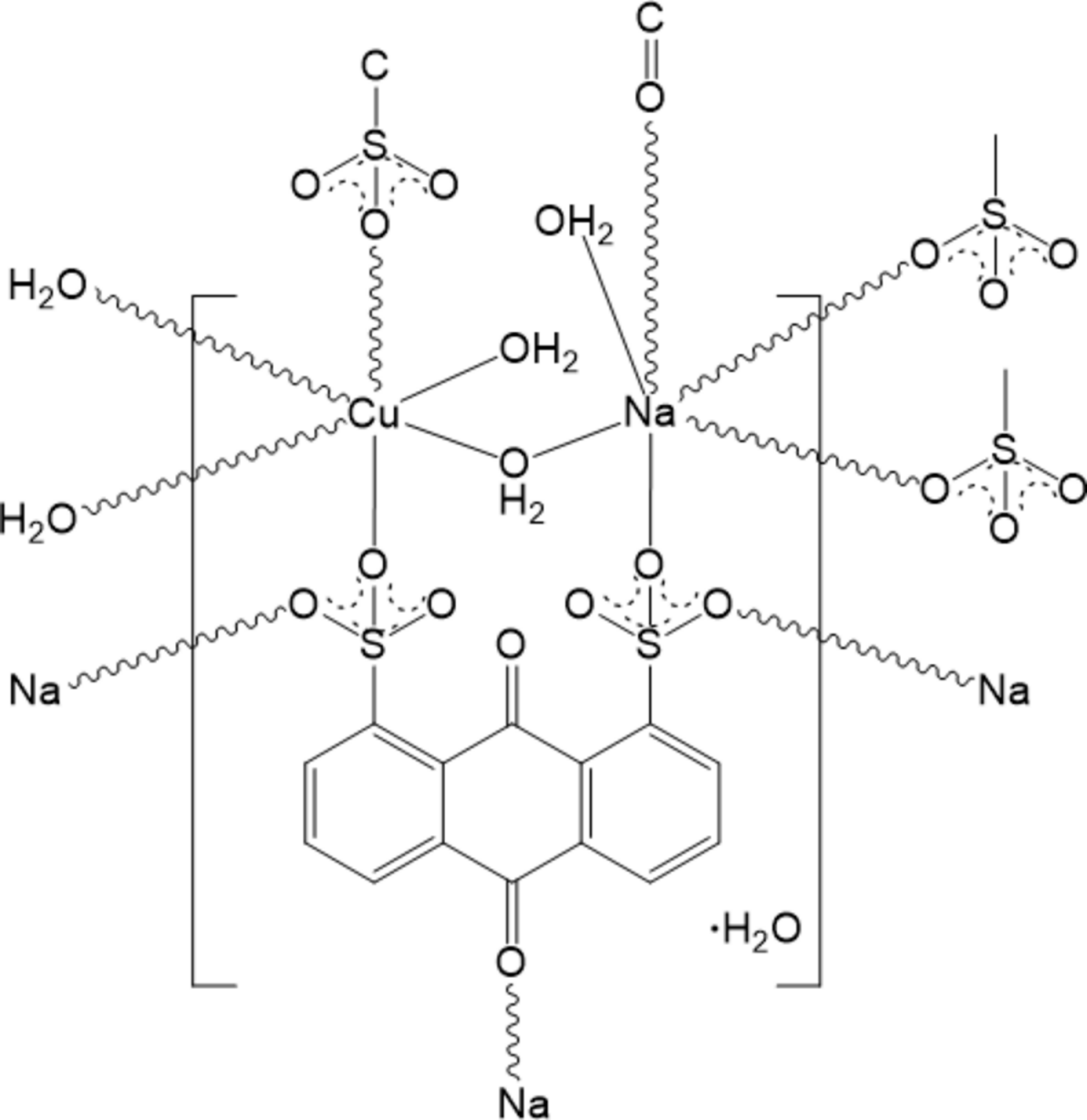


## 2. Structural commentary

**Figure 1:**
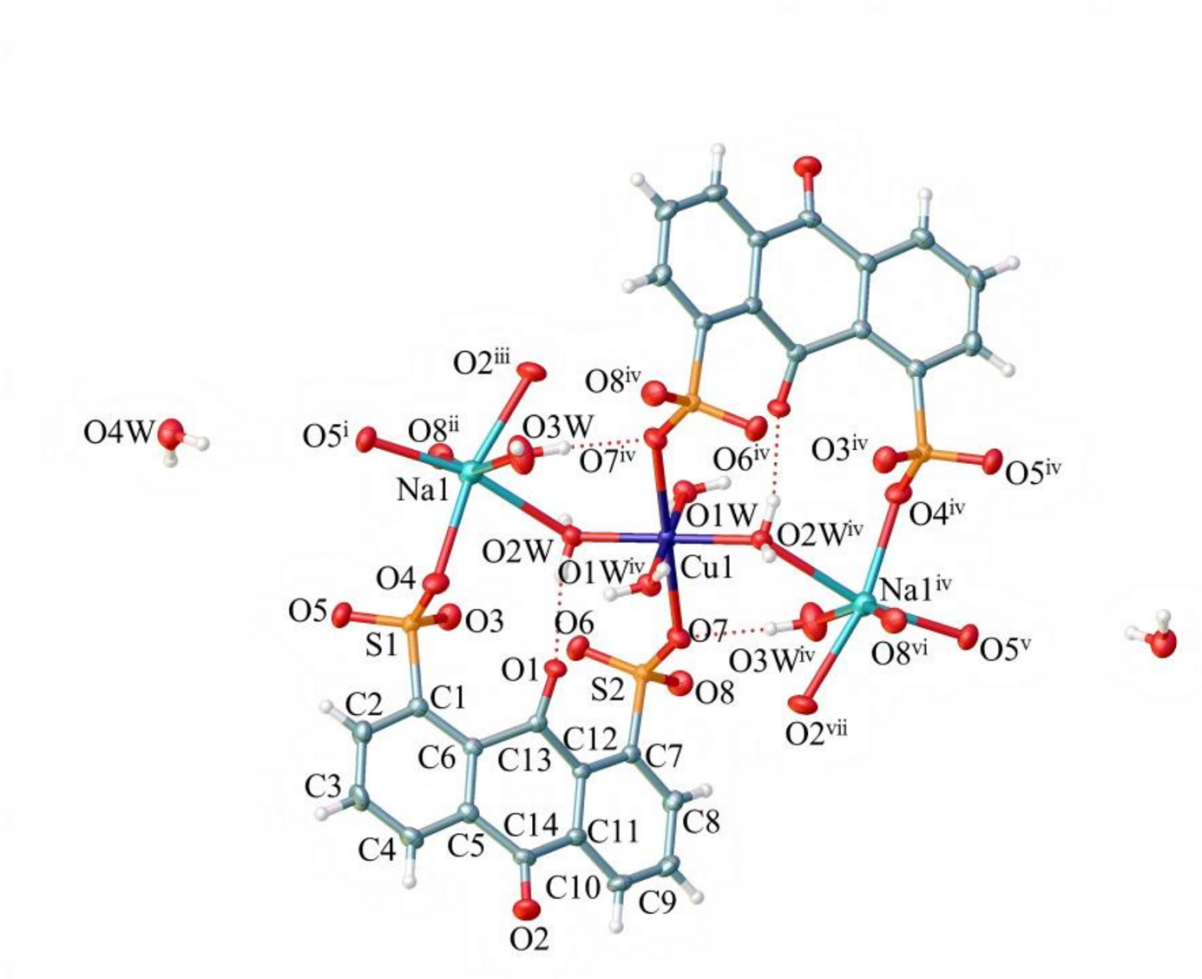
The coordination environment of the Cu^II^ atom in the title complex, showing 50% probability displacement ellipsoids. [Symmetry codes: (i) −*x* + 2, −*y* + 1, −*z* + 1; (ii) −*x* + 3, −*y* + 1, −*z* + 1; (iii) *x*, −*y* + 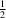, *z* + 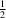; (iv) −*x* + 3, −*y*, −*z* + 1; (v) *x* + 1, *y* − 1, *z*; (vi) *x*, *y* − 1, *z*; (vii) –*x* + 3, *y* − 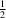, −*z* + 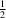.]

Complex **1** crystallizes in the centrosymmetric monoclinic space group *P*2_1_/*c*. The asymmetric unit comprises one sodium cation with a terminal aqua ligand, one half-occupied copper(II) cation with a terminal aqua ligand, one dianionic 1,8-AQDS^2−^ ligand, one bridging aqua ligand that bridges the sodium cation and the copper(II) cation, and one water mol­ecule of crystallization (Fig. 1 ▸).

In the crystal structure of **1**, the copper(II) atom resides on an inversion center, and adopts an distorted octa­hedral environment ascribed to the Jahn–Teller effect. The coordination sphere comprises a pair of terminal aqua ligands, a pair of bridging aqua ligands and a pair of O atoms from the sulfonate groups of two symmetry-related 1,8-AQDS^2−^ ligand. The Cu–water distances are similar, being 1.941 (1) Å for Cu1—O1*W* (terminal aqua ligand) and 2.005 (1) Å for Cu1—O2*W* (bridging aqua ligand). However, as a result of the Jahn–Teller effect, the Cu—O distances between Cu1 and the O atoms of sulfonate groups is 2.457 (1) Å, which is much longer compared to the Cu–water contacts. The non-linear O—Cu—O angles are in the range 88.66 (5) to 91.34 (5)°.

**Figure 2:**
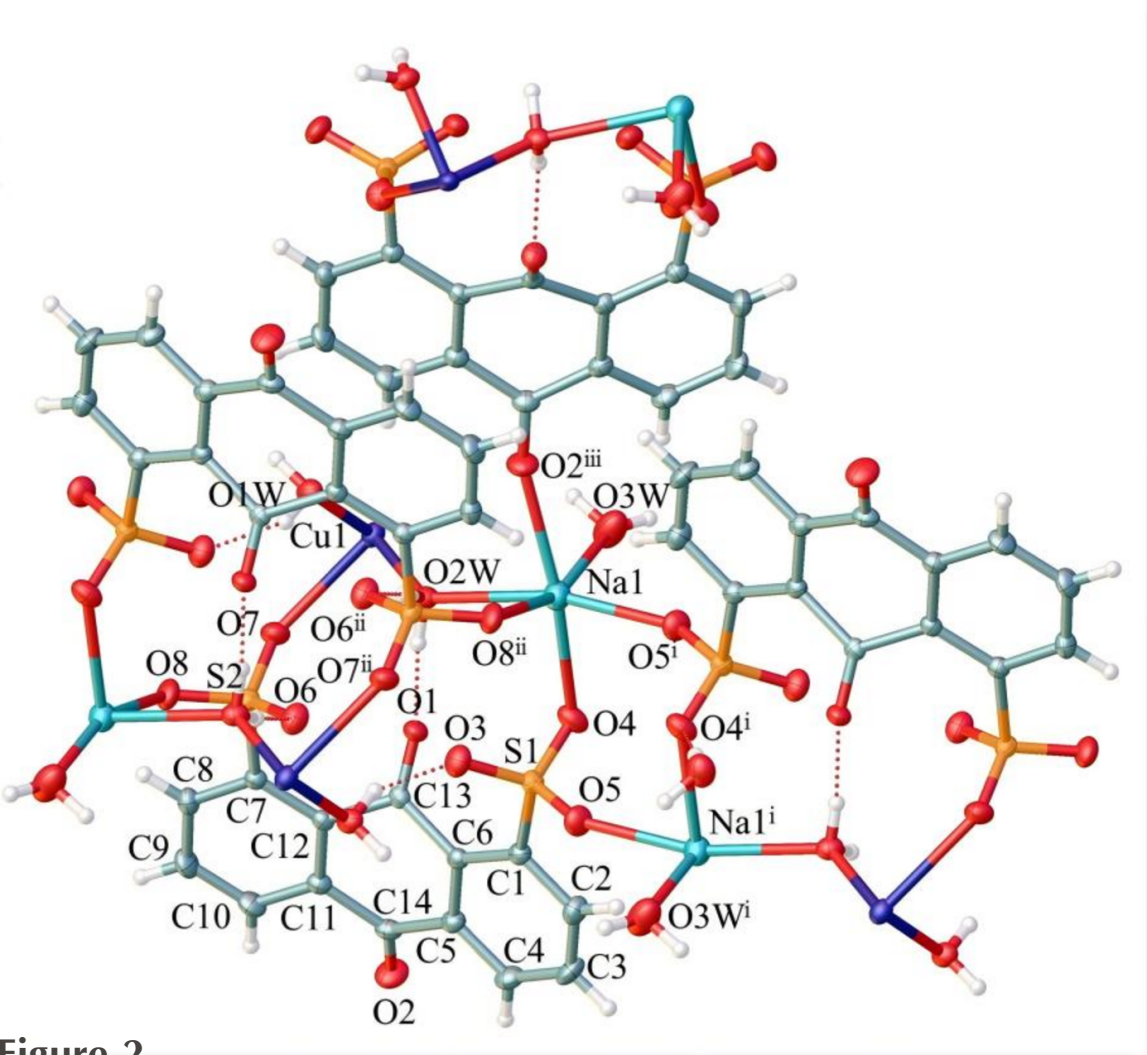
The coordination environment of the Na^I^ atom in the title complex, showing 50% probability displacement ellipsoids. [Symmetry codes: (i) −*x* + 2, −*y* + 1, −*z* + 1; (ii) −*x* + 3, −*y* + 1, −*z* + 1; (iii) *x*, −*y* + 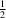, *z* + 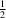.]

Each sodium cation in **1** is six-coordinated with a distorted octa­hedral environment constituted of one bridging aqua ligand, one terminal aqua ligand, three O atoms from sulfonate groups of three symmetry related 1,8-AQDS^2−^ ligands, and one O atom from the carbonyl group of another 1,8-AQDS^2−^ ligand (Fig. 2 ▸). The Na1—O3*W* (terminal aqua ligand) bond distance is 2.346 (2) Å, which is the shortest Na—O contact in the octa­hedral environment. The bond length Na1—O2*W* (bridging aqua ligand) is 2.620 (2) Å, representing the longest Na—O contact in this compound. It is inter­esting that the Na—O contacts in relation to the terminal or the bridging aqua ligands differs by 0.28 Å. Distances between Na1 and the three O atoms from three distinct sulfonate groups are: Na1—O4 = 2.497 (2) Å, Na1—O5^i^ = 2.378 (2) Å, and Na1—O8^ii^ = 2.418 (2) Å [symmetry codes: (i) −*x* + 2, −*y* + 1, −*z* + 1; (ii) −*x* + 3, −*y* + 1, −*z* + 1]. Bond length between Na and O from the anthra­quinone carbonyl group is Na1—O2^iii^ = 2.502 (2) Å [symmetry code: (iii) *x*, −*y* + 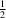, *z* + 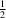). The non-linear O—Na—O angles fall in the range 82.19 (5) to 107.13 (6)°, indicating a more distorted octa­hedral coordination sphere as compared to that of Cu.

**Figure 3:**
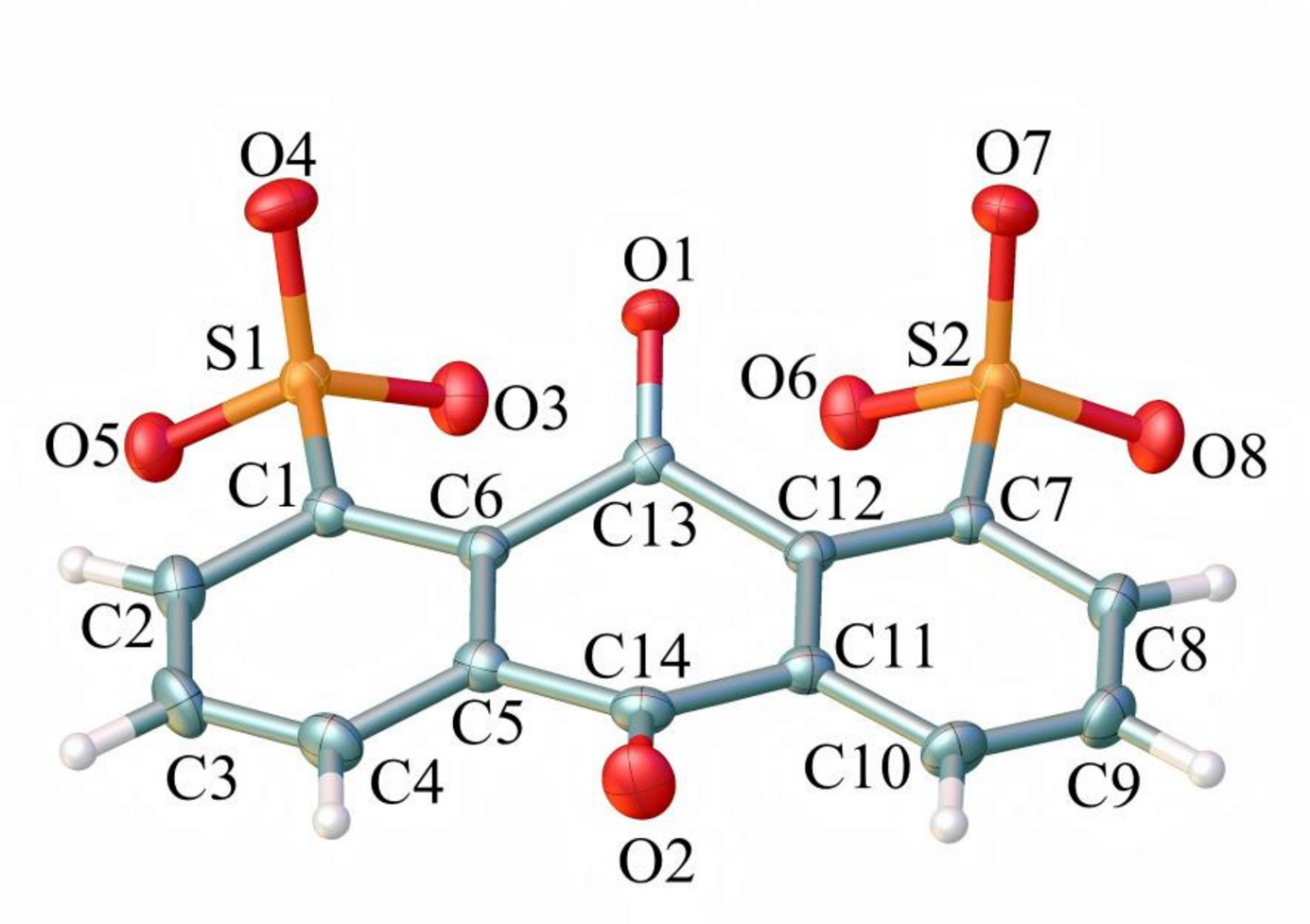
The butterfly conformation of the 1,8-AQDS^2−^ ligand in the title complex.

The 1,8-AQDS^2−^ ligand bears a complicated *μ*_5_-bridging coordination mode in this complex. Except for the coordinated sulfonate O atoms, one of the carbonyl group sitting opposite to the sulfonate groups also participates into coordination, which is a major factor that extends the layered structure into a 3-D coordination polymer. The 1,8-positioned bulky sulfonate groups exert a strong stereo resistance to the inter-positioned carbonyl group and lead to bending of 1,8-AQDS^2−^ ligand into a butterfly conformation. As shown in Fig. 3 ▸, the C5–C6–C11–C12 plane is defined as the basic plane (mean deviation 0.0007 Å). The dihedral angle between the C6–C13(=O1)–C12 plane and the basic plane is 28.8 (1)° while the C5–C14(=O2)–C11 plane subtends a dihedral angel of 14.3emsp14;(1)° with the basic plane. The two phenyl rings subtend dihedral angles of 8.9emsp14;(1)) and 9.5emsp14;(1)°, respectively, with the basic plane.

## 3. Supra­molecular features

**Figure 4:**
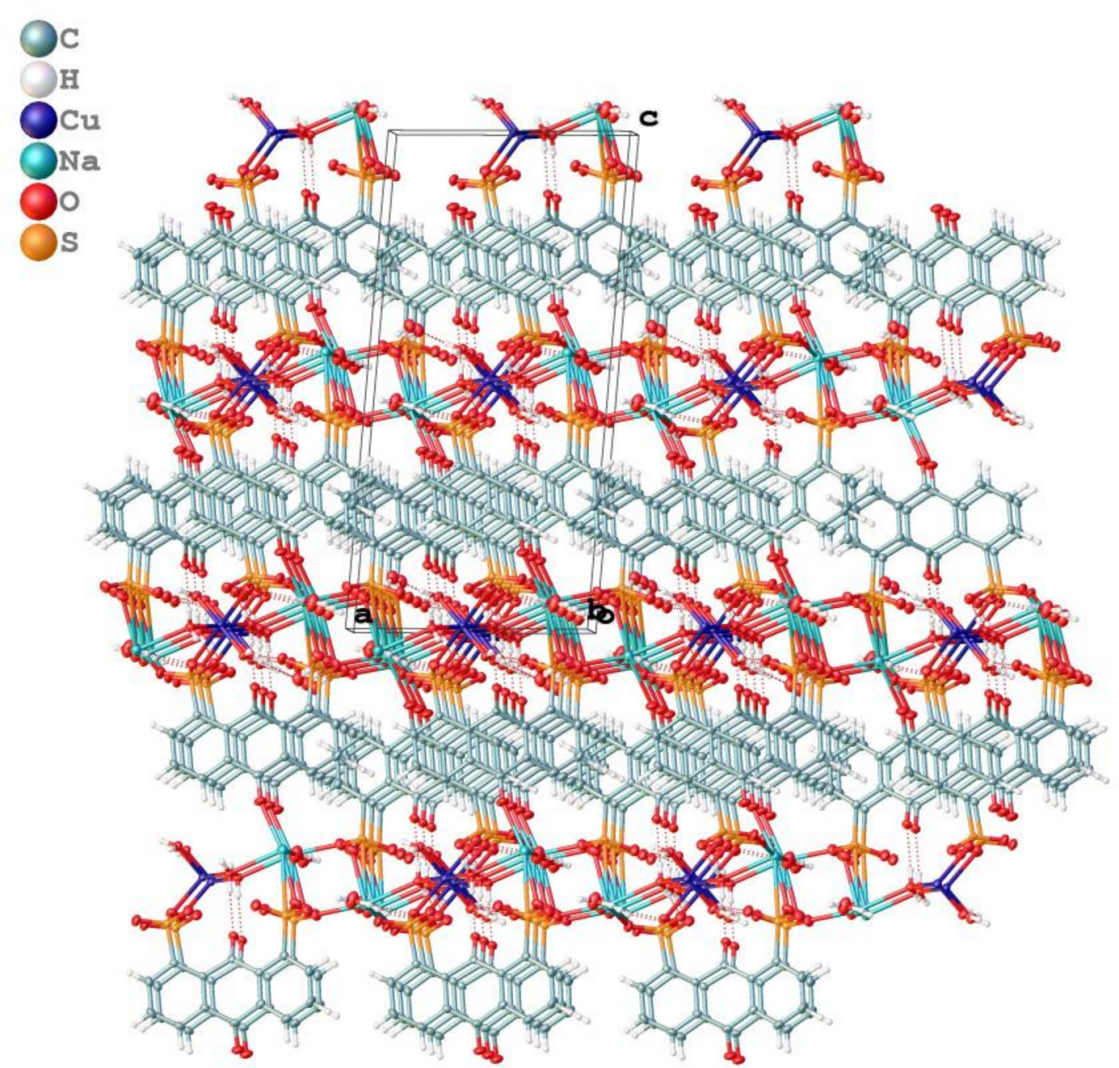
Packing along the *b* axis of the title complex.

Similar to that in many coordination complexes based on ligands containing sulfonate groups, the 3-D packing of complex **1** exhibits a pillar-layered framework, although the 2-D layers are actually linked together by Na–carbonyl coordination and not weak inter­actions as is usually the case (Fig. 4 ▸).

Weak inter­actions such as hydrogen bonding and π–π stacking do occur widely in the crystal packing. The aqua ligands are versatile hydrogen-bond donors and participate in a wide range of O—H⋯O hydrogen bonding with aqua-, sulfonate- and carbonyl-O atoms as acceptors. These O—H⋯O hydrogen bonds contribute differently in consolidating the 3-D framework (Table 1 ▸). A couple of π-π stacking inter­actions are observed between the C7–C12 phenyl rings of adjacent symmetry-related 1,8-AQDS^2−^ ligands, with plane-to-plane distances at 3.578 (1) Å and 3.580 (1) Å, respectively.

## 4. Thermal stability

Thermogravimetric analysis was performed using a crystalline sample of the title complex under an N_2_ atmosphere wherein the sample was heated to 800°C at a rate of 10°C min^−1^ (Figure S1). The complex starts to lose weight at 57°C, and the first stage weight loss corresponds to the loss of three of its four water mol­ecules (observed 11.0% *vs* calculated: 11.0%). It is believed that the solvent water mol­ecule and the two terminal aqua ligands are gone at this stage. The weight loss in the second stage (148–270°C) should then be ascribed to the loss of the bridging aqua ligand (observed 3.9% *vs* calculated: 3.7%), because the water mol­ecule that bridges Na and Cu centers should be more stable and will be the last one to be lost upon heating. At about 320°C, the ligand starts to decompose and loses one of its sulfonate groups to release SO_3_, which corresponds to stage III (observed 16.6% *vs* calculated: 16.2%) weight loss. Decomposition of the ligand continues with heating and with loss of the second sulfonate group it starts to release SO_3_ (430–600 °C), which corresponds to stage IV weight loss (observed 17.2% *vs* calculated: 16.2%).

## 5. Database survey

A search of the Cambridge Structural Database with WebCSD (https://www.ccdc.cam.ac.uk/structures/WebCSD; CSD version 5.43 with updats to November 2022; Groom *et al.*, 2016 ▸) revealed metal complexes of anthra­quinone-1,8-di­sulfonate (1,8-AQDS^2−^) remain unexplored to date.

## 6. Synthesis and crystallization

Under stirring, a 3 mL methanol solution of NaOH (8.0 mg, 0.2 mmol) was added into a 3 mL methanol solution of 1,8-H_2_AQDS (73.6 mg, 0.2 mmol), resulting in a white precipitate suspended in a brown–yellow solution. To the suspension were added 4 mL of Cu(NO_3_)_2_·3H_2_O (12.1 mg, 0.05 mmol) methanol solution. After stirring for 10 minutes, 2 mL of water were then added. A clear brown solution was obtained, which was stirred for another 10 minutes. After filtration, the solution was allowed to stand at room temperature for 2 days to give 52.5 mg (53.4% yield) of green prismatic crystals. IR(KBr, cm^−1^): 3604 (*w*), 3490 (*m*), 3421 (*s*), 3093 (*w*), 3018 (*w*), 1702 (*m*), 1679 (*m*), 1628 (*w*), 1572 (*w*), 1329 (*m*), 1242 (*m*), 1217 (*vs*), 1047 (*s*), 964 (*w*), 856 (*w*), 804 (*w*), 735 (*w*), 638 (*m*), 561 (*w*) cm^−1^.

## 7. Refinement

Crystal data, data collection and structure refinement details are summarized in Table 2 ▸. Aromatic H atoms were positioned geometrically and refined using a riding model, with *U*_iso_(H) = 1.2*U*_eq_(C). H atoms of water mol­ecules were found in difference-Fourier maps and refined using a riding model with fixed *U*_iso_(H) = 1.5*U*_eq_(O).

## Supplementary Material

Crystal structure: contains datablock(s) I. DOI: 10.1107/S2056989026003956/oi2034sup1.cif

Structure factors: contains datablock(s) I. DOI: 10.1107/S2056989026003956/oi2034Isup2.hkl

Supporting information file. DOI: 10.1107/S2056989026003956/oi2034Isup3.mol

Supporting information file. DOI: 10.1107/S2056989026003956/oi2034sup4.docx

CCDC reference: 2533984

Additional supporting information:  crystallographic information; 3D view; checkCIF report

Additional supporting information:  crystallographic information; 3D view; checkCIF report

## Figures and Tables

**Table 1:** Hydrogen-bond geometry (Å, °)

*D*—H⋯*A*	*D*—H	H⋯*A*	*D*⋯*A*	*D*—H⋯*A*
O1*W*—H1*WA*⋯O3^i^	0.84	1.95	2.737 (2)	155
O1*W*—H1*WB*⋯O4*W*^ii^	0.84	1.87	2.681 (2)	161
O2*W*—H2*WA*⋯O1	0.83	2.03	2.808 (2)	155
O2*W*—H2*WB*⋯O6^i^	0.87	1.80	2.669 (2)	175
O3*W*—H3*WA*⋯O7^iii^	0.91	2.12	3.008 (2)	165
O3*W*—H3*WB*⋯O4^iv^	0.83	2.29	3.093 (2)	165
O4*W*—H4*WA*⋯O5^v^	0.85	2.07	2.880 (2)	161
O4*W*—H4*WB*⋯O4^vi^	0.87	1.94	2.809 (2)	177

Symmetry codes: (i) 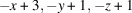; (ii) 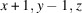; (iii) 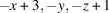; (iv) 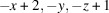; (v) 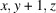; (vi) 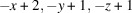.

**Table 2:** Experimental details

Crystal data	
Chemical formula	[Na_2_Cu(C_8_H_12_O_8_S_2_)_2_(H_2_O)_6_]·2(H_2_O)
*M*_r_	986.26
Crystal system, space group	Monoclinic, *P*2_1_/*c*
Temperature (K)	293
*a*, *b*, *c* (Å)	10.7839 (15), 7.1582 (10), 22.230 (3)
β (°)	94.661 (2)
*V* (Å^3^)	1710.3 (4)
*Z*	2
Radiation type	Mo *K*α
μ (mm^−1^)	1.01
Crystal size (mm)	0.4 × 0.3 × 0.2

Data collection	
Diffractometer	Bruker APEXII CCD
Absorption correction	Multi-scan (*SADABS*; Krause *et al.*, 2015 ▸)
*T*_min_, *T*_max_	0.615, 0.746
No. of measured, independent and observed [*I* > 2σ(*I*)] reflections	12719, 3511, 3137
*R*_int_	0.019
(sin θ/λ)_max_ (Å^−1^)	0.628

Refinement	
*R*[*F*^2^ > 2σ(*F*^2^)], *wR*(*F*^2^), *S*	0.025, 0.066, 1.07
No. of reflections	3511
No. of parameters	268
H-atom treatment	H-atom parameters constrained
Δρ_max_, Δρ_min_ (e Å^−3^)	0.36, −0.32

Computer programs: *APEX2* and *SAINT* (Bruker, 2014 ▸), *SHELXT2014/5* (Sheldrick, 2015*a* ▸), *SHELXL2018/3* (Sheldrick, 2015*b* ▸) and *OLEX2* (Dolomanov *et al.*, 2009 ▸).

## supplementary crystallographic information

### Poly[[di-µ2-aqua-κ4O:O-tetraaquabis(µ5-anthraquinone-1,8-disulfonato-κ5O:O':O'':O''':O'''')copper(II)disodium] dihydrate] Crystal data

**Table:**

[Na_2_Cu(C_8_H_12_O_8_S_2_)_2_(H_2_O)_6_]·2(H_2_O)	*F*(000) = 1006
*M**_r_* = 986.26	*D*_x_ = 1.915 Mg m^−^^3^
Monoclinic, *P*2_1_/*c*	Mo *K*α radiation, λ = 0.71073 Å
*a* = 10.7839 (15) Å	Cell parameters from 7185 reflections
*b* = 7.1582 (10) Å	θ = 2.5–27.6°
*c* = 22.230 (3) Å	µ = 1.01 mm^−^^1^
β = 94.661 (2)°	*T* = 293 K
*V* = 1710.3 (4) Å^3^	Prism, brownish green
*Z* = 2	0.4 × 0.3 × 0.2 mm

### Poly[[di-µ2-aqua-κ4O:O-tetraaquabis(µ5-anthraquinone-1,8-disulfonato-κ5O:O':O'':O''':O'''')copper(II)disodium] dihydrate] Data collection

**Table:**

Bruker APEXII CCD diffractometer	3137 reflections with *I* > 2σ(*I*)
φ and ω scans	*R*_int_ = 0.019
Absorption correction: multi-scan (SADABS; Krause *et al.*, 2015)	θ_max_ = 26.5°, θ_min_ = 1.8°
*T*_min_ = 0.615, *T*_max_ = 0.746	*h* = −13→13
12719 measured reflections	*k* = −8→8
3511 independent reflections	*l* = −27→27

### Poly[[di-µ2-aqua-κ4O:O-tetraaquabis(µ5-anthraquinone-1,8-disulfonato-κ5O:O':O'':O''':O'''')copper(II)disodium] dihydrate] Refinement

**Table:**

Refinement on *F*^2^	0 restraints
Least-squares matrix: full	Hydrogen site location: mixed
*R*[*F*^2^ > 2σ(*F*^2^)] = 0.025	H-atom parameters constrained
*wR*(*F*^2^) = 0.066	*w* = 1/[σ^2^(*F*_o_^2^) + (0.0251*P*)^2^ + 1.5553*P*] where *P* = (*F*_o_^2^ + 2*F*_c_^2^)/3
*S* = 1.07	(Δ/σ)_max_ = 0.001
3511 reflections	Δρ_max_ = 0.36 e Å^−^^3^
268 parameters	Δρ_min_ = −0.32 e Å^−^^3^

### Poly[[di-µ2-aqua-κ4O:O-tetraaquabis(µ5-anthraquinone-1,8-disulfonato-κ5O:O':O'':O''':O'''')copper(II)disodium] dihydrate] Special details

**Table:**

Geometry. All esds (except the esd in the dihedral angle between two l.s. planes) are estimated using the full covariance matrix. The cell esds are taken into account individually in the estimation of esds in distances, angles and torsion angles; correlations between esds in cell parameters are only used when they are defined by crystal symmetry. An approximate (isotropic) treatment of cell esds is used for estimating esds involving l.s. planes.

### Poly[[di-µ2-aqua-κ4O:O-tetraaquabis(µ5-anthraquinone-1,8-disulfonato-κ5O:O':O'':O''':O'''')copper(II)disodium] dihydrate] Fractional atomic coordinates and isotropic or equivalent isotropic displacement parameters (Å^2^)

**Table:**

	*x*	*y*	*z*	*U*_iso_*/*U*_eq_	
Cu1	1.500000	0.000000	0.500000	0.01836 (9)	
Na1	1.17167 (7)	0.27300 (12)	0.55206 (4)	0.02836 (18)	
S1	1.12707 (4)	0.49651 (6)	0.41893 (2)	0.01815 (11)	
S2	1.61805 (4)	0.36474 (6)	0.40306 (2)	0.01789 (10)	
O1	1.34403 (12)	0.25015 (18)	0.37928 (6)	0.0212 (3)	
O2	1.28269 (14)	0.3207 (2)	0.15062 (6)	0.0318 (3)	
O3	1.25186 (12)	0.5553 (2)	0.43879 (6)	0.0272 (3)	
O4	1.09581 (14)	0.31577 (19)	0.44381 (6)	0.0282 (3)	
O5	0.96636 (13)	0.3612 (2)	0.57200 (6)	0.0274 (3)	
O6	1.53097 (12)	0.49328 (19)	0.42803 (6)	0.0259 (3)	
O7	1.60938 (13)	0.17394 (19)	0.42504 (6)	0.0262 (3)	
O8	1.74520 (12)	0.4340 (2)	0.40842 (6)	0.0283 (3)	
C1	1.11995 (16)	0.4698 (2)	0.33793 (8)	0.0182 (4)	
C2	1.00742 (18)	0.5216 (3)	0.30740 (9)	0.0254 (4)	
H2	0.941808	0.558345	0.329377	0.030*	
C3	0.99110 (19)	0.5194 (3)	0.24496 (10)	0.0284 (4)	
H3	0.914624	0.552458	0.225523	0.034*	
C4	1.08776 (18)	0.4685 (3)	0.21157 (9)	0.0250 (4)	
H4	1.077422	0.469503	0.169630	0.030*	
C5	1.20137 (17)	0.4153 (3)	0.24117 (8)	0.0194 (4)	
C6	1.21934 (16)	0.4138 (2)	0.30450 (8)	0.0165 (3)	
C7	1.57528 (16)	0.3555 (2)	0.32329 (8)	0.0163 (3)	
C8	1.67307 (17)	0.3567 (3)	0.28607 (8)	0.0219 (4)	
H8	1.754512	0.358796	0.303396	0.026*	
C9	1.65155 (18)	0.3549 (3)	0.22356 (9)	0.0262 (4)	
H9	1.718182	0.354014	0.199502	0.031*	
C10	1.53143 (18)	0.3544 (3)	0.19733 (8)	0.0248 (4)	
H10	1.516653	0.352687	0.155533	0.030*	
C11	1.43214 (17)	0.3566 (2)	0.23367 (8)	0.0183 (4)	
C12	1.45180 (16)	0.3551 (2)	0.29703 (8)	0.0157 (3)	
C13	1.33986 (16)	0.3370 (2)	0.33240 (8)	0.0152 (3)	
C14	1.30373 (17)	0.3591 (3)	0.20396 (8)	0.0203 (4)	
O1W	1.61332 (12)	0.12057 (18)	0.56030 (6)	0.0219 (3)	
H1WA	1.635769	0.230912	0.553858	0.033*	
H1WB	1.681069	0.067212	0.571258	0.033*	
O2W	1.38383 (12)	0.21707 (18)	0.50523 (6)	0.0208 (3)	
H2WA	1.374857	0.261596	0.470484	0.031*	
H2WB	1.415457	0.307086	0.527904	0.031*	
O3W	1.12642 (16)	−0.0474 (2)	0.54618 (9)	0.0457 (4)	
H3WA	1.200286	−0.106327	0.555140	0.069*	
H3WB	1.072886	−0.130517	0.543740	0.069*	
O4W	0.84187 (14)	1.0273 (2)	0.60719 (7)	0.0322 (3)	
H4WA	0.882629	1.109742	0.590029	0.048*	
H4WB	0.860629	0.918542	0.592529	0.048*	

### Poly[[di-µ2-aqua-κ4O:O-tetraaquabis(µ5-anthraquinone-1,8-disulfonato-κ5O:O':O'':O''':O'''')copper(II)disodium] dihydrate] Atomic displacement parameters (Å^2^)

**Table:**

	*U*^11^	*U*^22^	*U*^33^	*U*^12^	*U*^13^	*U*^23^
Cu1	0.01744 (16)	0.01871 (16)	0.01847 (16)	0.00042 (12)	−0.00143 (12)	−0.00487 (12)
Na1	0.0252 (4)	0.0331 (4)	0.0267 (4)	−0.0017 (3)	0.0020 (3)	−0.0016 (3)
S1	0.0185 (2)	0.0177 (2)	0.0187 (2)	0.00024 (16)	0.00391 (17)	−0.00137 (17)
S2	0.0164 (2)	0.0208 (2)	0.0162 (2)	0.00011 (17)	−0.00034 (16)	−0.00061 (17)
O1	0.0216 (6)	0.0250 (7)	0.0172 (6)	−0.0008 (5)	0.0025 (5)	0.0054 (5)
O2	0.0350 (8)	0.0427 (9)	0.0167 (7)	0.0025 (7)	−0.0033 (6)	−0.0059 (6)
O3	0.0225 (7)	0.0290 (7)	0.0296 (7)	−0.0029 (6)	−0.0012 (6)	−0.0076 (6)
O4	0.0369 (8)	0.0225 (7)	0.0264 (7)	−0.0025 (6)	0.0103 (6)	0.0031 (6)
O5	0.0258 (7)	0.0265 (7)	0.0307 (8)	0.0060 (6)	0.0071 (6)	−0.0037 (6)
O6	0.0237 (7)	0.0285 (7)	0.0253 (7)	0.0018 (6)	0.0006 (6)	−0.0097 (6)
O7	0.0292 (7)	0.0249 (7)	0.0243 (7)	0.0022 (6)	0.0014 (6)	0.0070 (6)
O8	0.0181 (7)	0.0380 (8)	0.0280 (7)	−0.0052 (6)	−0.0029 (5)	−0.0022 (6)
C1	0.0185 (9)	0.0174 (9)	0.0185 (9)	−0.0013 (7)	0.0003 (7)	0.0002 (7)
C2	0.0190 (9)	0.0282 (10)	0.0288 (10)	0.0036 (8)	0.0011 (8)	−0.0024 (8)
C3	0.0201 (9)	0.0337 (11)	0.0297 (11)	0.0064 (8)	−0.0079 (8)	0.0005 (9)
C4	0.0265 (10)	0.0277 (10)	0.0196 (9)	0.0002 (8)	−0.0057 (8)	0.0012 (8)
C5	0.0204 (9)	0.0183 (9)	0.0190 (9)	−0.0018 (7)	−0.0016 (7)	0.0000 (7)
C6	0.0172 (8)	0.0148 (8)	0.0173 (8)	−0.0022 (7)	0.0005 (7)	0.0009 (7)
C7	0.0182 (8)	0.0146 (8)	0.0160 (8)	−0.0004 (7)	0.0015 (7)	−0.0002 (7)
C8	0.0179 (9)	0.0236 (9)	0.0247 (10)	−0.0015 (7)	0.0049 (7)	−0.0007 (8)
C9	0.0241 (10)	0.0312 (11)	0.0249 (10)	0.0000 (8)	0.0116 (8)	−0.0004 (8)
C10	0.0303 (10)	0.0290 (10)	0.0159 (9)	−0.0009 (8)	0.0072 (8)	0.0006 (8)
C11	0.0224 (9)	0.0170 (9)	0.0157 (8)	0.0001 (7)	0.0023 (7)	0.0004 (7)
C12	0.0186 (8)	0.0129 (8)	0.0157 (8)	−0.0003 (6)	0.0033 (7)	0.0006 (6)
C13	0.0172 (8)	0.0133 (8)	0.0150 (8)	−0.0029 (6)	0.0010 (6)	−0.0028 (6)
C14	0.0266 (10)	0.0195 (9)	0.0144 (8)	−0.0011 (7)	−0.0002 (7)	0.0017 (7)
O1W	0.0233 (7)	0.0196 (7)	0.0222 (7)	−0.0035 (5)	−0.0031 (5)	−0.0008 (5)
O2W	0.0246 (7)	0.0201 (6)	0.0173 (6)	0.0013 (5)	−0.0010 (5)	−0.0009 (5)
O3W	0.0344 (9)	0.0316 (9)	0.0691 (12)	0.0011 (7)	−0.0075 (8)	0.0013 (8)
O4W	0.0309 (8)	0.0251 (8)	0.0410 (9)	−0.0022 (6)	0.0060 (7)	−0.0016 (6)

### Poly[[di-µ2-aqua-κ4O:O-tetraaquabis(µ5-anthraquinone-1,8-disulfonato-κ5O:O':O'':O''':O'''')copper(II)disodium] dihydrate] Geometric parameters (Å, º)

**Table:**

Cu1—O1W	1.9413 (12)	C3—C4	1.377 (3)
Cu1—O1W^i^	1.9414 (12)	C4—H4	0.9300
Cu1—O2W	2.0053 (13)	C4—C5	1.396 (3)
Cu1—O2W^i^	2.0053 (13)	C5—C6	1.406 (2)
Na1—S1	3.3643 (10)	C5—C14	1.487 (3)
Na1—O2^ii^	2.5016 (16)	C6—C13	1.498 (2)
Na1—O4	2.4968 (16)	C7—C8	1.392 (2)
Na1—O5	2.3783 (15)	C7—C12	1.410 (2)
Na1—O8^iii^	2.4176 (17)	C8—H8	0.9300
Na1—O2W	2.6202 (15)	C8—C9	1.390 (3)
Na1—O3W	2.3463 (19)	C9—H9	0.9300
S1—O3	1.4443 (14)	C9—C10	1.377 (3)
S1—O4	1.4573 (14)	C10—H10	0.9300
S1—O5^iv^	1.4582 (14)	C10—C11	1.393 (3)
S1—C1	1.8061 (19)	C11—C12	1.407 (2)
S2—O6	1.4563 (14)	C11—C14	1.485 (3)
S2—O7	1.4561 (14)	C12—C13	1.499 (2)
S2—O8	1.4540 (14)	O1W—H1WA	0.8418
S2—C7	1.7970 (18)	O1W—H1WB	0.8427
O1—C13	1.211 (2)	O2W—H2WA	0.8340
O2—C14	1.220 (2)	O2W—H2WB	0.8704
C1—C2	1.392 (3)	O3W—H3WA	0.9090
C1—C6	1.411 (2)	O3W—H3WB	0.8277
C2—H2	0.9300	O4W—H4WA	0.8451
C2—C3	1.385 (3)	O4W—H4WB	0.8740
C3—H3	0.9300		
			
O1W—Cu1—O1W^i^	180.0	C3—C2—C1	121.38 (18)
O1W—Cu1—O2W^i^	91.34 (5)	C3—C2—H2	119.3
O1W^i^—Cu1—O2W^i^	88.66 (5)	C2—C3—H3	119.9
O1W—Cu1—O2W	88.66 (5)	C4—C3—C2	120.20 (18)
O1W^i^—Cu1—O2W	91.34 (5)	C4—C3—H3	119.9
O2W—Cu1—O2W^i^	180.0	C3—C4—H4	120.3
O2^ii^—Na1—S1	157.23 (5)	C3—C4—C5	119.48 (18)
O2^ii^—Na1—O2W	86.32 (5)	C5—C4—H4	120.3
O4—Na1—S1	23.31 (3)	C4—C5—C6	121.23 (17)
O4—Na1—O2^ii^	166.67 (6)	C4—C5—C14	118.32 (17)
O4—Na1—O2W	82.19 (5)	C6—C5—C14	120.45 (16)
O5—Na1—S1	88.23 (4)	C1—C6—C13	123.82 (16)
O5—Na1—O2^ii^	107.13 (6)	C5—C6—C1	118.46 (16)
O5—Na1—O4	84.84 (5)	C5—C6—C13	117.50 (15)
O5—Na1—O8^iii^	91.46 (6)	C8—C7—S2	116.11 (14)
O5—Na1—O2W	166.11 (6)	C8—C7—C12	119.31 (16)
O8^iii^—Na1—S1	85.95 (4)	C12—C7—S2	124.52 (13)
O8^iii^—Na1—O2^ii^	77.14 (5)	C7—C8—H8	119.3
O8^iii^—Na1—O4	109.06 (6)	C9—C8—C7	121.39 (17)
O8^iii^—Na1—O2W	88.11 (5)	C9—C8—H8	119.3
O2W—Na1—S1	77.89 (3)	C8—C9—H9	120.1
O3W—Na1—S1	113.68 (5)	C10—C9—C8	119.89 (17)
O3W—Na1—O2^ii^	82.48 (6)	C10—C9—H9	120.1
O3W—Na1—O4	90.90 (6)	C9—C10—H10	120.1
O3W—Na1—O5	94.43 (6)	C9—C10—C11	119.71 (18)
O3W—Na1—O8^iii^	159.63 (7)	C11—C10—H10	120.1
O3W—Na1—O2W	90.74 (6)	C10—C11—C12	121.33 (17)
O3—S1—Na1	78.85 (6)	C10—C11—C14	118.38 (16)
O3—S1—O4	112.41 (9)	C12—C11—C14	120.29 (16)
O3—S1—O5^iv^	113.04 (8)	C7—C12—C13	123.84 (15)
O3—S1—C1	107.37 (8)	C11—C12—C7	118.35 (15)
O4—S1—Na1	42.69 (6)	C11—C12—C13	117.61 (15)
O4—S1—O5^iv^	112.58 (8)	O1—C13—C6	120.98 (15)
O4—S1—C1	106.97 (8)	O1—C13—C12	121.37 (16)
O5^iv^—S1—Na1	105.17 (6)	C6—C13—C12	117.36 (15)
O5^iv^—S1—C1	103.76 (8)	O2—C14—C5	120.88 (17)
C1—S1—Na1	144.90 (6)	O2—C14—C11	121.49 (17)
O6—S2—C7	106.12 (8)	C11—C14—C5	117.60 (15)
O7—S2—O6	113.82 (8)	Cu1—O1W—H1WA	118.3
O7—S2—C7	106.15 (8)	Cu1—O1W—H1WB	118.7
O8—S2—O6	112.72 (9)	H1WA—O1W—H1WB	102.7
O8—S2—O7	112.23 (9)	Cu1—O2W—Na1	135.34 (6)
O8—S2—C7	104.96 (8)	Cu1—O2W—H2WA	105.6
C14—O2—Na1^v^	161.68 (14)	Cu1—O2W—H2WB	113.0
S1—O4—Na1	114.00 (8)	Na1—O2W—H2WA	105.7
S1^iv^—O5—Na1	151.07 (9)	Na1—O2W—H2WB	88.3
S2—O8—Na1^iii^	130.17 (9)	H2WA—O2W—H2WB	105.3
C2—C1—S1	115.02 (14)	Na1—O3W—H3WA	105.5
C2—C1—C6	119.23 (17)	Na1—O3W—H3WB	147.9
C6—C1—S1	125.63 (14)	H3WA—O3W—H3WB	106.0
C1—C2—H2	119.3	H4WA—O4W—H4WB	108.0
			
Na1—S1—C1—C2	120.74 (14)	C3—C4—C5—C6	−0.4 (3)
Na1—S1—C1—C6	−63.5 (2)	C3—C4—C5—C14	179.39 (18)
Na1^v^—O2—C14—C5	−21.0 (6)	C4—C5—C6—C1	−0.8 (3)
Na1^v^—O2—C14—C11	160.7 (3)	C4—C5—C6—C13	174.14 (17)
S1—C1—C2—C3	176.00 (16)	C4—C5—C14—O2	−15.8 (3)
S1—C1—C6—C5	−174.63 (13)	C4—C5—C14—C11	162.54 (17)
S1—C1—C6—C13	10.8 (3)	C5—C6—C13—O1	−144.69 (17)
S2—C7—C8—C9	−178.31 (15)	C5—C6—C13—C12	29.3 (2)
S2—C7—C12—C11	176.94 (13)	C6—C1—C2—C3	−0.1 (3)
S2—C7—C12—C13	−8.4 (3)	C6—C5—C14—O2	163.99 (18)
O3—S1—O4—Na1	40.93 (10)	C6—C5—C14—C11	−17.7 (3)
O3—S1—C1—C2	−143.94 (15)	C7—S2—O8—Na1^iii^	112.45 (11)
O3—S1—C1—C6	31.86 (18)	C7—C8—C9—C10	0.9 (3)
O4—S1—C1—C2	95.20 (15)	C7—C12—C13—O1	−30.2 (3)
O4—S1—C1—C6	−89.01 (17)	C7—C12—C13—C6	155.84 (16)
O5^iv^—S1—O4—Na1	−88.11 (10)	C8—C7—C12—C11	−0.2 (3)
O5^iv^—S1—C1—C2	−24.02 (16)	C8—C7—C12—C13	174.46 (16)
O5^iv^—S1—C1—C6	151.77 (16)	C8—C9—C10—C11	0.3 (3)
O6—S2—O8—Na1^iii^	−2.60 (14)	C9—C10—C11—C12	−1.4 (3)
O6—S2—C7—C8	138.21 (14)	C9—C10—C11—C14	179.05 (18)
O6—S2—C7—C12	−39.03 (17)	C10—C11—C12—C7	1.4 (3)
O7—S2—O8—Na1^iii^	−132.70 (10)	C10—C11—C12—C13	−173.61 (17)
O7—S2—C7—C8	−100.37 (15)	C10—C11—C14—O2	15.4 (3)
O7—S2—C7—C12	82.39 (16)	C10—C11—C14—C5	−162.97 (17)
O8—S2—C7—C8	18.65 (17)	C11—C12—C13—O1	144.51 (17)
O8—S2—C7—C12	−158.59 (15)	C11—C12—C13—C6	−29.5 (2)
C1—S1—O4—Na1	158.55 (8)	C12—C7—C8—C9	−0.9 (3)
C1—C2—C3—C4	−1.1 (3)	C12—C11—C14—O2	−164.15 (18)
C1—C6—C13—O1	29.9 (3)	C12—C11—C14—C5	17.5 (3)
C1—C6—C13—C12	−156.08 (16)	C14—C5—C6—C1	179.44 (16)
C2—C1—C6—C5	1.0 (3)	C14—C5—C6—C13	−5.6 (2)
C2—C1—C6—C13	−173.57 (17)	C14—C11—C12—C7	−179.11 (16)
C2—C3—C4—C5	1.4 (3)	C14—C11—C12—C13	5.9 (2)

Symmetry codes: (i) −*x*+3, −*y*, −*z*+1; (ii) *x*, −*y*+1/2, *z*+1/2; (iii) −*x*+3, −*y*+1, −*z*+1; (iv) −*x*+2, −*y*+1, −*z*+1; (v) *x*, −*y*+1/2, *z*−1/2.

### Poly[[di-µ2-aqua-κ4O:O-tetraaquabis(µ5-anthraquinone-1,8-disulfonato-κ5O:O':O'':O''':O'''')copper(II)disodium] dihydrate] Hydrogen-bond geometry (Å, º)

**Table:**

*D*—H···*A*	*D*—H	H···*A*	*D*···*A*	*D*—H···*A*
O1*W*—H1*WA*···O3^iii^	0.84	1.95	2.737 (2)	155
O1*W*—H1*WB*···O4*W*^vi^	0.84	1.87	2.681 (2)	161
O2*W*—H2*WA*···O1	0.83	2.03	2.808 (2)	155
O2*W*—H2*WB*···O6^iii^	0.87	1.80	2.669 (2)	175
O3*W*—H3*WA*···O7^i^	0.91	2.12	3.008 (2)	165
O3*W*—H3*WB*···O4^vii^	0.83	2.29	3.093 (2)	165
O4*W*—H4*WA*···O5^viii^	0.85	2.07	2.880 (2)	161
O4*W*—H4*WB*···O4^iv^	0.87	1.94	2.809 (2)	177

Symmetry codes: (i) −*x*+3, −*y*, −*z*+1; (iii) −*x*+3, −*y*+1, −*z*+1; (iv) −*x*+2, −*y*+1, −*z*+1; (vi) *x*+1, *y*−1, *z*; (vii) −*x*+2, −*y*, −*z*+1; (viii) *x*, *y*+1, *z*.
