# Supplementary material for: Synthesis and crystal structure of a mixed-metal 3D coordination polymer poly[[bis­(μ5-anthra­quinone-1,8-di­sulfonato-κ5O:O′:O′′:O′′′:O′′′′)di-μ2-aqua-κ4O:O-tetra­aqua­copper(II)disodium] dihydrate]
Source: Acta Crystallogr E Crystallogr Commun. 2026 May 15;82(Pt 6):678–82. doi: 10.1107/S2056989026003956 (PMC13238972; doi:10.1107/S2056989026003956)
Supplement: Supplementary file 4 [file e-82-00678-sup4.docx]

Supporting information

**Synthesis and crystal structure of a mix-metal 3-D coordination polymer *catena*-poly[[(bis-*μ*_2_-aqua-*κ*^2^O:O)[bis(*μ*_5_-anthraquinone-1,8-disulfonato-*κ*^5^O:O':O'':O''':O'''')] [bis(aquasodium)][(bisaqua)copper(II)]]dihydrate]**

Xuan-Yi Chen, Jia Wei, Juan He, Hui-Lei Gao, Xu-Dong Chen, Gan Xu*


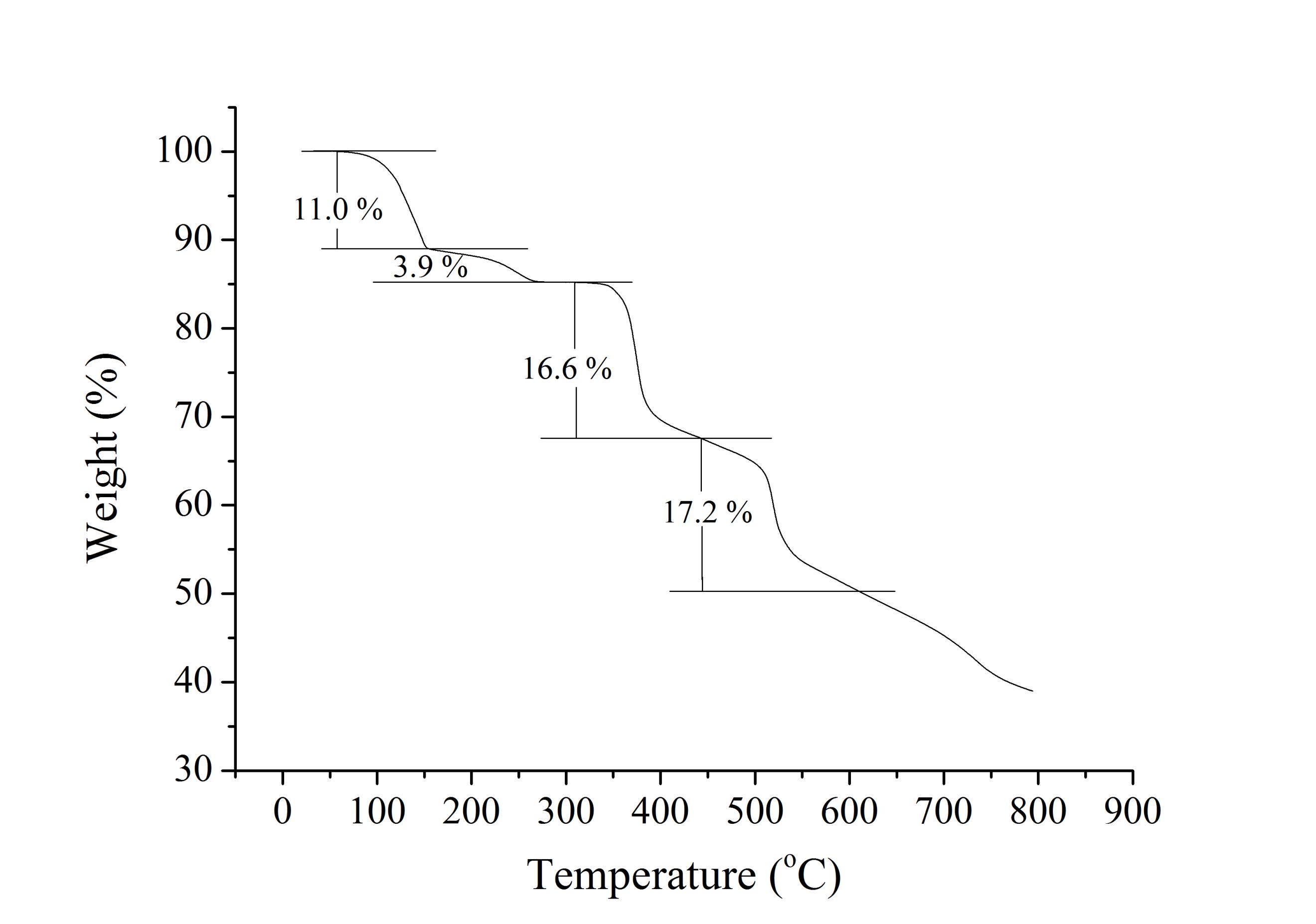


**Figure S1**

Thermogravimetric analysis of the title complex.

Computer detail

Data collection: APEX2 (Bruker, 2014); cell refinement: SAINT (Bruker, 2013); data reduction: SAINT (Bruker, 2013); program(s) used to solve structure: SHELXT (Sheldrick, 2015); program(s) used to refine structure: SHELXL2013 (Sheldrick, 2015); molecular graphics: OLEX2 (Dolomanov et al., 2009); software used to prepare material for publication: publCIF (Westrip, 2010).

*Catena*-poly[[(bis-*μ*_2_-aqua-*κ*^2^O:O)[bis(*μ*_5_-anthraquinone-1,8-disulfonato-*κ*^5^O:O':O'':O''':O'''')] [bis(aquasodium)][(bisaqua)copper(II)]]dihydrate]

| Crystal data |  | |
| --- | --- | --- |
| [CuNa_2_(C_14_H_6_O_8_S_2_)_2_(H_2_O)_6_]·H_2_O |  | *F*(000) = 1006 |
| *M*_r_ = 986.26 |  | *Dx* = 1.915 Mg m^−3^ |
| Monoclinic, *P*2_1/_*n* |  | Mo *Kα* radiation, *λ* = 0.71073 Å |
| *a =* 10.7839 (2) Å |  | Cell parameters from 7185reflections |
| *b* = 7.1582 (1) Å |  | *θ* = 2.5-27.6° |
| *c =* 22.230 (3) Å |  | *μ* = 1.01 mm^−1^ |
| *β* = 94.661 (2)^o^ |  | *T* = 293 K |
| *V* = 1710.3(4) Å^3^ |  | Prism, brownish green |
| *Z*= 2 |  | 0.40×0.30×0.20 mm |
|  |  |  |
| *Data collection* | | |
| Bruker APEXII CCD area-detector  diffractometer |  | 12719 measured reflections |
|  |  | 3511 independent reflections |
| Radiation source: fine-focus sealed tube |  | 3137 reflections with *I* > 2*σ*(*I*) |
|  |  | *R*_int_ = 0.019 |
| phi and omega scans |  | *θ*_max_ = 26.5°, *θ*_min_ = 1.8° |
| Absorption correction: multi-scan  SADABS |  | *h* = -13→13 |
|  |  | *k* = -8→8 |
| *T*min = 0.647, *T*max = 0.781 |  | *l* = -27→27 |
|  |  |  |
|  |  |  |
| *Refinement* | | |
| Refinement on *F*^2^ |  | Hydrogen site location mixed |
| Least-squares matrix: full |  |  |
| *R*[*F*^2^ > *2σ*(*F*^2^)] = 0.025 |  | H-atom parameters constrained |
| *wR*(*F^2^*) = 0.066 |  | *w* = 1/[*σ*^2^(*F*_o_^2^)+(0.0251*P*)^2^+1.555*P*] where *P* = (*F*_o_^2^+2*F*_c_^2^)/^3^ |
| *S =* 1.070 |  |  |
| 3511 reflections |  | (Δ/*σ*)max = 0.001 |
| 268 parameters |  | Δ*ρ*_max_ =0.36 e/Å^3^ |
| 0 restraints |  | Δ*ρ*_min_ = -0.32 e/Å^3^ |
|  |  |  |

*Special details*

**Geometry.** All esds (except the esd in the dihedral angle between two l.s. planes) are estimated using the full covariance matrix. The cell esds are taken into account individually in the estimation of esds in distances, angles and torsion angles; correlations between esds in cell parameters are only used when they are defined by crystal symmetry. An approximate (isotropic) treatment of cell esds is used for estimating esds involving l.s. planes.

*Fractional atomic coordinates and isotropic or equivalent isotropic displacement parameters (Å^2^)*

|  | *x* | *y* | *z* | U(eq) |
| --- | --- | --- | --- | --- |
| Na1 | 11716.7(7) | 2730.0(12) | 5520.6(4) | 28.36(18) |
| Cu1 | 15000 | 0 | 5000 | 18.36(9) |
| S1 | 11270.7(4) | 4965.1(6) | 4189.3(2) | 18.15(11) |
| S2 | 16180.5(4) | 3647.4(6) | 4030.6(2) | 17.89(10) |
| O1 | 13440.3(12) | 2501.5(18) | 3792.8(6) | 21.2(3) |
| O2 | 12826.9(14) | 3207(2) | 1506.2(6) | 31.8(3) |
| O3 | 12518.6(12) | 5553(2) | 4387.9(6) | 27.2(3) |
| O4 | 10958.1(14) | 3157.7(19) | 4438.1(6) | 28.2(3) |
| O5 | 10336.4(13) | 6388(2) | 4280.0(6) | 27.4(3) |
| O6 | 15309.7(12) | 4932.8(19) | 4280.3(6) | 25.9(3) |
| O7 | 16093.8(13) | 1739.4(19) | 4250.4(6) | 26.2(3) |
| O8 | 17452.0(12) | 4340(2) | 4084.2(6) | 28.3(3) |
| C1 | 11199.5(16) | 4698(2) | 3379.3(8) | 18.2(4) |
| C2 | 10074.2(18) | 5216(3) | 3074.0(9) | 25.4(4) |
| C3 | 9911.0(19) | 5194(3) | 2449.6(10) | 28.4(4) |
| C4 | 10877.6(18) | 4685(3) | 2115.7(9) | 25.0(4) |
| C5 | 12013.7(17) | 4153(3) | 2411.7(8) | 19.4(4) |
| C6 | 12193.4(16) | 4138(2) | 3045.0(8) | 16.5(3) |
| C7 | 15752.8(16) | 3555(2) | 3232.9(8) | 16.3(3) |
| C8 | 16730.7(17) | 3567(3) | 2860.7(8) | 21.9(4) |
| C9 | 16515.5(18) | 3549(3) | 2235.6(9) | 26.2(4) |
| C10 | 15314.3(18) | 3544(3) | 1973.3(8) | 24.8(4) |
| C11 | 14321.4(17) | 3566(2) | 2336.7(8) | 18.3(4) |
| C12 | 14518.0(16) | 3551(2) | 2970.3(8) | 15.7(3) |
| C13 | 13398.6(16) | 3370(2) | 3324.0(8) | 15.2(3) |
| C14 | 13037.3(17) | 3591(3) | 2039.6(8) | 20.3(4) |
| O1W | 16133.2(12) | 1205.7(18) | 5603.0(6) | 21.9(3) |
| O2W | 13838.3(12) | 2170.7(18) | 5052.3(6) | 20.8(3) |
| O3W | 11264.2(16) | -474(2) | 5461.8(9) | 45.7(4) |
| O4W | 8418.7(14) | 10273(2) | 6071.9(7) | 32.2(3 |
|  |  |  |  |  |

*Atomic displacement parameters (Å^2^)*

|  | U11 | U22 | U33 | U23 | U13 | U12 |
| --- | --- | --- | --- | --- | --- | --- |
| Na1 | 25.2(4) | 33.1(4) | 26.7(4) | -1.6(3) | 2.0(3) | -1.7(3) |
| Cu1 | 17.44(16) | 18.71(16) | 18.47(16) | -4.87(12) | -1.43(12) | 0.42(12) |
| S1 | 18.5(2) | 17.7(2) | 18.7(2) | -1.37(17) | 3.91(17) | 0.24(16) |
| S2 | 16.4(2) | 20.8(2) | 16.2(2) | -0.61(17) | -0.34(16) | 0.11(17) |
| O1 | 21.6(6) | 25.0(7) | 17.2(6) | 5.4(5) | 2.5(5) | -0.8(5) |
| O2 | 35.0(8) | 42.7(9) | 16.7(7) | -5.9(6) | -3.3(6) | 2.5(7) |
| O3 | 22.5(7) | 29.0(7) | 29.6(7) | -7.6(6) | -1.2(6) | -2.9(6) |
| O4 | 36.9(8) | 22.5(7) | 26.4(7) | 3.1(6) | 10.3(6) | -2.5(6) |
| O5 | 25.8(7) | 26.5(7) | 30.7(8) | -3.7(6) | 7.1(6) | 6.0(6) |
| O6 | 23.7(7) | 28.5(7) | 25.3(7) | -9.7(6) | 0.6(6) | 1.8(6) |
| O7 | 29.2(7) | 24.9(7) | 24.3(7) | 7.0(6) | 1.4(6) | 2.2(6) |
| O8 | 18.1(7) | 38.0(8) | 28.0(7) | -2.2(6) | -2.9(5) | -5.2(6) |
| C1 | 18.5(8) | 17.4(9) | 18.5(9) | 0.2(7) | 0.3(7) | -1.3(7) |
| C2 | 19.0(9) | 28.2(10) | 28.8(10) | -2.4(8) | 1.1(8) | 3.6(8) |
| C3 | 20.1(9) | 33.7(11) | 29.7(11) | 0.5(9) | -7.9(8) | 6.4(8) |
| C4 | 26.5(10) | 27.7(10) | 19.6(9) | 1.2(8) | -5.7(8) | 0.2(8) |
| C5 | 20.4(9) | 18.3(9) | 19.0(9) | 0.0(7) | -1.6(7) | -1.8(7) |
| C6 | 17.2(8) | 14.8(8) | 17.3(8) | 0.9(7) | 0.5(7) | -2.2(7) |
| C7 | 18.2(8) | 14.6(8) | 16.0(8) | -0.2(7) | 1.5(7) | -0.4(7) |
| C8 | 17.9(9) | 23.6(9) | 24.7(10) | -0.7(8) | 4.9(7) | -1.5(7) |
| C9 | 24.1(10) | 31.2(11) | 24.9(10) | -0.4(8) | 11.6(8) | 0.0(8) |
| C10 | 30.3(10) | 29(1) | 15.9(9) | 0.6(8) | 7.2(8) | -0.9(8) |
| C11 | 22.4(9) | 17.0(9) | 15.7(8) | 0.4(7) | 2.3(7) | 0.1(7) |
| C12 | 18.6(8) | 12.9(8) | 15.7(8) | 0.6(6) | 3.3(7) | -0.3(6) |
| C13 | 17.2(8) | 13.3(8) | 15.0(8) | -2.8(6) | 1.0(6) | -2.9(6) |
| C14 | 26.6(10) | 19.5(9) | 14.4(8) | 1.7(7) | -0.2(7) | -1.1(7) |
| O1W | 23.3(7) | 19.6(7) | 22.2(7) | -0.8(5) | -3.1(5) | -3.5(5) |
| O2W | 24.6(7) | 20.1(6) | 17.3(6) | -0.9(5) | -1.0(5) | 1.3(5) |
| O3W | 34.4(9) | 31.6(9) | 69.1(12) | 1.3(8) | -7.5(8) | 1.1(7) |
| O4W | 30.9(8) | 25.1(8) | 41.0(9) | -1.6(6) | 6.0(7) | -2.2(6) |

*Geometric parameters (Å, º)*

| Na1-S1 | 3.3643(10) |  | O1-C13 | 1.211(2) |
| --- | --- | --- | --- | --- |
| Na1-O2^1^ | 2.5016(16) |  | O2-Na1^5^ | 2.5015(16) |
| Na1-O4 | 2.4968(16) |  | O2-C14 | 1.220(2) |
| Na1-O5^2^ | 2.3783(15) |  | O5-Na1^2^ | 2.3783(15) |
| Na1-O8^3^ | 2.4176(17) |  | O8-Na1^3^ | 2.4176(17) |
| Na1-O2W | 2.6202(15) |  | C1-C2 | 1.392(3) |
| Na1-O3W | 2.3463(19) |  | C1-C6 | 1.411(2) |
| Cu1-O7 | 2.4572(13) |  | C2-C3 | 1.385(3) |
| Cu1-O7^4^ | 2.4572(13) |  | C3-C4 | 1.377(3) |
| Cu1-O1W | 1.9413(12) |  | C4-C5 | 1.396(3) |
| Cu1-O1W^4^ | 1.9414(12) |  | C5-C6 | 1.406(2) |
| Cu1-O2W | 2.0053(13) |  | C5-C14 | 1.487(3) |
| Cu1-O2W^4^ | 2.0053(13) |  | C6-C13 | 1.498(2) |
| S1-O3 | 1.4443(14) |  | C7-C8 | 1.392(2) |
| S1-O4 | 1.4573(14) |  | C7-C12 | 1.410(2) |
| S1-O5 | 1.4583(14) |  | C8-C9 | 1.390(3) |
| S1-C1 | 1.8061(19) |  | C9-C10 | 1.377(3) |
| S2 -O6 | 1.4563(14) |  | C10-C11 | 1.393(3) |
| S2-O7 | 1.4561(14) |  | C11-C12 | 1.407(2) |
| S2-O8 | 1.4540(14) |  | C11-C14 | 1.485(3) |
| S2 -C7 | 1.7970(18) |  | C12-C13 | 1.499(2) |

*Hydrogen-bond geometry (Å, º)*

| D−H···*A* | D−H | H···*A* | D···*A* | D−H···*A* |
| --- | --- | --- | --- | --- |
| O1*W*−H1*W*A···O3^i^ | 0.84 | 1.95 | 2.737(1) | 155 |
| O1*W*−H1*W*B···O4W^ii^ | 0.84 | 1.87 | 2.681(1) | 161 |
| O2*W*−H2*W*A···O1 | 0.83 | 2.03 | 2.808(1) | 155 |
| O2*W*−H2*W*B···O6^i^ | 0.87 | 1.80 | 2.669(1) | 175 |
| O3*W*−H3*W*A···O7^iii^ | 0.91 | 2.12 | 3.009(1) | 165 |
| O3*W*−H3*W*B···O4^iv^ | 0.83 | 2.29 | 3.094(1) | 165 |
| O4*W*−H4*W*A···O5^v^ | 0.85 | 2.07 | 2.880(1) | 161 |
| O4*W*−H4*W*B···O4^vi^ | 0.87 | 1.94 | 2.809(1) | 177 |

Symmetry codes: (i) −*x*+3, −*y*+1, −*z*+1; (ii) *x*+1, *y*−1, *z*; (iii) −*x*+3, −*y*, −*z*+1; (iv) −*x*+2, −*y*, −*z*+1; (v) *x*, *y*+1, *z*; (vi) −*x*+2, −*y*+1, −*z*+1.
